# Naotaifang extract treatment results in increased ferroportin expression in the hippocampus of rats subjected to cerebral ischemia

**DOI:** 10.3892/mmr.2015.3309

**Published:** 2015-02-06

**Authors:** JUN LIAO, XING XIA, GUO-ZUO WANG, YONG-MEI SHI, JIN-WEN GE

**Affiliations:** 1 Department of Anatomy, Hunan University of Chinese Medicine, Changsha, Hunan 410208, P.R. China; 2 Campus Network Center, Hunan University of Chinese Medicine, Changsha, Hunan 410208, P.R. China; 3 College of Integrated Traditional Chinese and Western Medicine, Hunan University of Chinese Medicine, Changsha, Hunan 410208, P.R. China

**Keywords:** ferroportin, naotaifang extract, cerebral ischemia

## Abstract

The expression of Ferroportin (Fpn) was examined at different time points in rats following focal cerebral ischemia treated with or without the traditional Chinese medicine Naotaifang. Initially, rats were randomly divided into 2, 6, 12, 24 and 72 h groups following middle cerebral artery occlusion (MCAO) and the mRNA and protein level of Fpn was detected by immunohistochemistry and reverse transcription polymerase chain reaction (RT-PCR) at the above time points. Secondly, the rats were randomly divided into five groups as follows: Sham surgery group, model group, low-dose group (3 g/kg NTE), medium dose group (9 g/kg NTE) and the high-dose group (27 g/kg NTE). After 3 days of corresponding therapy by intragastric administration once a day, the regional cerebral ischemia model was reproduced by the MCAO suture method. On the third day, the neurological behavior of the rats was analyzed by neurobehavioral assessment. Fpn in the hippocampal CA2 region was measured by immunohistochemistry and the mRNA level of Fpn was detected by RT-PCR. Expression of Fpn in the hippocampal CA2 region reached a peak 12 h after surgery (P<0.05, compared with the model group). The high-dose group (27 g/kg NTE) exhibited a lower neurological behavior score (P<0.05) and a higher level of expression of Fpn at the mRNA and protein level compared with the sham surgery group and model group (P<0.05). Dysregulation of intracellular iron balance is possibly a new mechanism underlying cerebral ischemia. NTE can protect the neuronal population in the hippocampal CA2 region by adjusting the expression of Fpn to balance iron levels following cerebral ischemia.

## Introduction

Cerebral ischemia is a condition in which there is insufficient blood flow to the brain caused by cerebral vasospasm or an embolism. Previous studies have demonstrated that cerebral ischemia triggers a cascade of pathophysiological events, including glutamate-dependent excitotoxicity, calcium overload, apoptosis, inflammation, free radical formation, nitric oxide production and mitochondrial damage, leading to neuronal cell death (1–5). Through investigating the mechanism underlying cerebral ischemic injury, a theoretical basis for clinical treatment can be proposed and may have far-reaching significance for the prevention and treatment of cerebral ischemia (6).

Iron is an essential trace element in the human body. It is involved in synthesis of the myelin sheath and neurotransmission. Iron is also a type of catalyst, which increases the concentration of reactive oxygen species. The increased production of reactive oxygen species and lipid peroxidation damages neurons in cerebral ischemia (7,8). Cerebral hypoxia leads to iron accumulation and lipid peroxidation in oligodendrocytes in one-day old Wistar rats during development (7). Whether iron accumulation and overload in neurons following cerebral ischemic injury is a novel mechanism requires further investigation.

Iron is composed of heme and non-heme iron in the human body (9). Heme iron is required for the synthesis of heme. Intracellular heme iron can increase heme oxygenase 1 expression and lead to oxidative stress and cell membrane damage (10). Non-heme iron is involved in cell respiration and catalyzing antibody production (11). Serum ferritin is a marker of the body’s non-heme iron store (12). Hepcidin is secreted by the liver and is important in the regulation of iron transport (13). Mutation of the gene BCS1L leads to a mitochondrial disorder and elevated serum ferritin (14). Accumulation of iron is observed in various neurodegenerative disorders, including Alzheimer’s and Parkinson’s disease (15). Excess iron increases ROS expression and activates the caspase protein family, which contributes to apoptosis. The presence of excess iron is therefore recognized as a major risk factor for neurodegenerative diseases (16–17). Previous studies have demonstrated that patients undergoing transfusion therapy are at risk of iron overload with associated tissue damage (15–17). Hyperferritinemia and increased iron stores are associated with the severity of liver damage in non-alcoholic fatty liver disease (18). Iron overload and oxidative stress are involved in the endometriosis-associated inflammatory reaction (19). Certain characteristics of metabolic syndrome are mainly attributed to iron overload (20). As a type of non-heme iron export protein (21–23), ferroportin (Fpn) is abundant in the small intestine and macrophages (24). Previous studies demonstrated that Fpn is also expressed in the hippocampus, cerebral cortex, thalamus, brainstem and cerebellum (25,26). Hepcidin binds to Fpn and promotes its internalization and degradation. Fpn disease, the most common non-HFE hereditary iron-loading disorder, is caused by a loss of iron export function of Fpn resulting in early and preferential iron accumulation in kupffer cells and macrophages (27). Schulz *et al* found that astrocytes can secrete Fpn to promote remyelination following axonal injury (28). Certain studies have proposed that inflammatory cytokines alter the expression of Fpn resulting in iron accumulation (29). Fpn can therefore respond to intraneural non-heme iron metabolism in cerebral ischemia.

Traditional Chinese medicine has made certain achievements for cerebral ischemia (30–32). Naotaifang extract (NTE) is an extract of a traditional Chinese medicine compound, which improves blood circulation. A previous study demonstrated that NTE is clinically effective for the treatment of cerebral ischemia and the therapeutic mechanism includes anticoagulation and angiogenesis (33).

In order to reveal the dysregulation of intracellular iron and examine the mechanisms underlying cerebral brain, the present study investigated the expression of Fpn in the hippocampal CA2 region cells following induction of cerebral ischemia in rats treated with NTE.

## Materials and methods

### Animals

A total of 100 healthy adult male Sprague Dawley rats weighing 220–250 g (SPF grade) were provided by the Experimental Animal Center of Hunan University of Chinese Medicine (Changsha, China). The study was approved by the ethics committee of the College of Traditional Chinese Medicine (Changsha, China).

### Drugs

NTE consists of astragalus root, chuanxiong and dilong. The extract was acquired by water decoction and alcohol extraction. The active ingredients include astragaloside, ligustrazine and ferulic acid (extracted by pharmaceutical preparation at the Department of Hunan Traditional Chinese Medicine University) mixed with physiological saline to achieve the required concentration.

### Experiment one

A total of 50 healthy male Sprague Dawley rats were assigned to the 2 h group (n=10), 6 h group (n=10), 12 h group (n=10), 24 h group (n=10) and 72 h group (n=10) using a random digits table. The rats were used to establish the middle cerebral artery occlusion (MCAO) model.

### Experiment two

A total of 50 healthy male Sprague Dawley rats were assigned to either the sham surgery group (n=10) or the surgery group (n=40) using a random digits table. The rats in the surgery group were used to establish the MCAO model. According to the postoperative treatment, the surgery group was divided into the model group (0.9% NaCL), low-dose group (3 g/g NTE), medium dose group (9 g/kg NTE) and high-dose group (27 g/kg NTE). Each group was treated with the corresponding dose through intragastric administration for the following three days after surgery. The animal specimens were collected 72 h after surgery.

### Animal modeling

Focal cerebral ischemia was induced by intra-arterial suture occlusion of the right middle cerebral artery (MCA) (34). MCAO was induced by using the intraluminal filament technique. Right common and external carotid arteries were ligated and the internal carotid artery was closed. A fish wire (d=0.28 mm) was advanced through the right internal carotid artery to the origin of the MCA. The sham group was treated identically, with the exception that no intraluminal filament was insert into the MCA. Neurological assessment was used to confirm successful MCAO. Following surgery (35), the cerebral cortex and hippocampus CA2 area exhibited marked neuronal damage on the right side.

### Neurobehavioral assessment

Neurobehavioral scores were assessed in each animal following the final treatment at 72 h. A modification of a previous method was used to evaluate the neurological deficit (36). The five categories of motor neurological findings were scored: 0, no observable symptom; 1, contralateral forelimb flexion; 2, contralateral circling; 3, tumble contralateral side; 4, unable to walk, loss of consciousness.

### Preparation of tissue slices

All rats were perfused with paraformaldehyde (4%; Sigma-Aldrich, St. Louis, MO, USA) under anesthesia and fixed at different time points (2, 6, 12, 24 and 72 h) following brain ischemia. The brains were then removed and fixed for 1 h, washed with sodium chloride (Sigma-Aldrich), dehydrated with gradient alcohol (Sigma-Aldrich), embedded in paraffin (Sigma-Aldrich) and sectioned in 4 *μ*m thick coronal sections.

### Immunohistochemical staining and image analysis

Paraffin sections were incubated at 60°C for 30 min, then dewaxed in xylene (Sigma-Aldrich) and gradient alcohol. Slides were soaked in a solution of 3% H_2_O_2_ (Sigma-Aldrich) for 10 min at room temperature to block endogenous peroxidases. Rabbit-anti-rat Fpn monoclonal antibody (1:100; Proteintech, Chicago, IL, USA) was added and incubated for 2 h at 37°C. The sections were washed three times in 0.01 mol/l phosphate-buffered saline (PBS; Sigma-Aldrich) for 5 min. The sections were placed in wet boxes and incubated for 40 min at 37°C with polyclonal biotin-labeled goat anti-rabbit IgG (cat no. PV-600; Proteintech). Subsequently, the sections were washed twice in PBS for 5 min and stained with 3,3′-diaminobenzidine (Sigma-Aldrich). The sections were then observed under an optical microscope (Olympus, Tokyo, Japan). Five sections were randomly selected from each slice. Image analysis was performed using Image Pro Plus 5.0 software (Media Cybernetics, Inc., Rockville, MD, USA) to determine the integral optical density (IOD) of Fpn positive areas in each visual field (magnification, ×400) and an average was calculated.

### Expression of Fpn mRNA detected by RT-PCR

At different time points following surgery or 72 h after NTE treatment, 100 mg tissue was extracted from the hippocampus using 1 ml TRIzol (Invitrogen Life Technologies, Carlsbad, CA, USA). RNA was reverse transcribed in a final volume of 10 *μ*l containing 1 *μg* of total RNA, 1 *μ*l oligo (dT), 10 mM of each deoxyribonucleoside triphosphate (2 *μ*l), 20 units RNasin, 200 units AMV Reverse Transcriptase and 4 *μ*l of 5X Reverse Transcriptase buffer with diethylpyrocarbonate H_2_O (Reverse-transcription kit, Invitrogen Life Technologies). PCR was performed using 3 *μ*l of synthesized cDNA with 10 *μ*l 2X PCR mix, 1 *μ*l of each primer, 4 *μ*l of PCR buffer and ddH_2_O to give a total reaction volume of 20 *μ*l. All common components were added to a master mix (Takara, Dalian, China) and then aliquoted. The cycling conditions were as follows: Initial denaturation at 94°C for 4 min followed by 28 cycles of 94°C for 15 sec, 56°C for 30 sec, 72°C for 30 sec and a final extension at 72°C for 5 min. The primers of Fpn were designed (Table I) and Actin was used as a loading control.

### Statistical analysis

All data are expressed as the mean ± standard deviation and were statistically analyzed using SPSS 11.0 software (SPSS, Inc., Chicago, IL, USA). The two-sample t-test was used for comparison among groups. P<0.05 was considered to indicate a statistically significant difference.

## Results

### Experiment one

**Figure 1:**
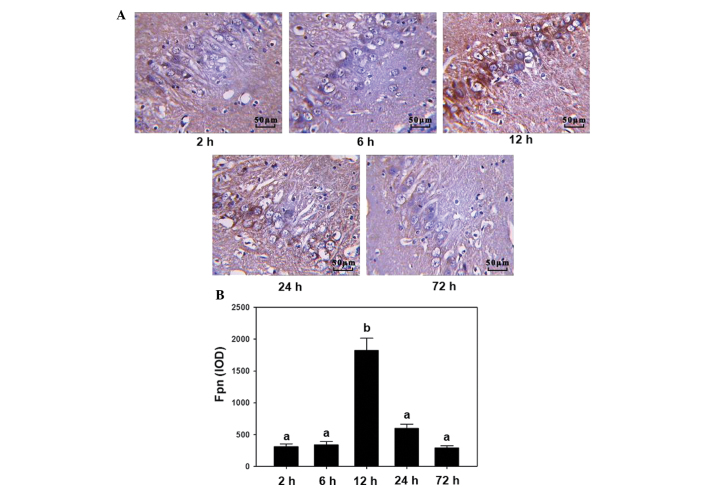
Fpn expression in the hippocampal CA2 region at different time points detected by immunohistochemistry. (A) Immunohistochemistry results at 2, 6, 12, 24 and 72 h. (B) IOD of Fpn among all the groups. P<0.05 for comparisons between bars labelled with different letters. Fpn, ferroportin; IOD, integral optical density.

**Figure 2:**
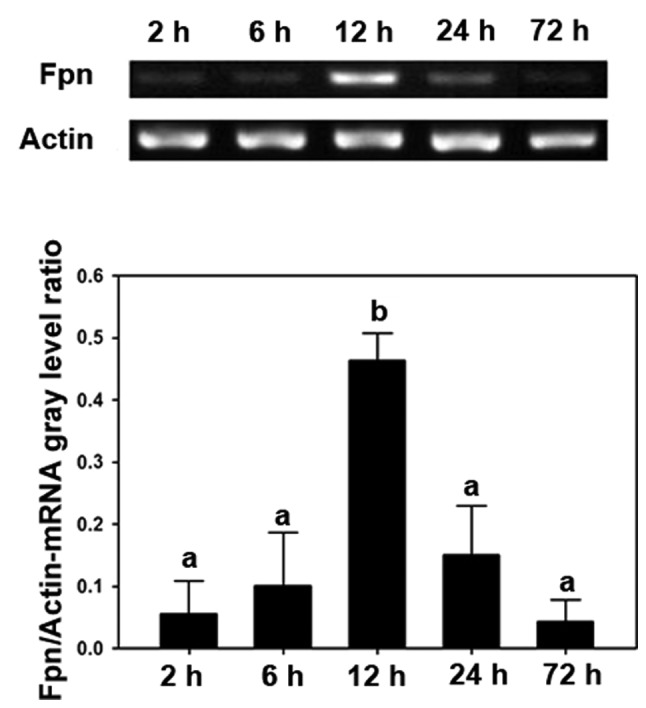
Fpn expression in the hippocampal CA2 region at different time points by RT-PCR. (A) RT-PCR results at 2, 6, 12, 24 and 72 h. (B) Relative expression of Fpn among all the groups. P<0.05 for comparisons between bars labelled with different letters. Fpn, ferroportin; RT-PCR, reverse transcription polymerase chain reaction.

The expression of Fpn was detected using immunohistochemistry and RT-PCR in the hippocampal CA2 region, at different time points following induction of cerebral ischemia. The results from the immunohistochemistry experiments showed that the darkest staining was observed at 12 h while the changes were less marked in the 2, 6, 24 and 72 h groups (Fig. 1A). RT-PCR demonstrated similar results; after 12 h treatment, the expression of Fpn was significantly increased compared with the time points (P<0.05; Fig. 1B). The RT-PCR analysis also supported the results that only the 12 h treatment group demonstrated a significant increase among all the groups (P<0.05; Fig. 2).

### Experiment two

**Figure 3:**
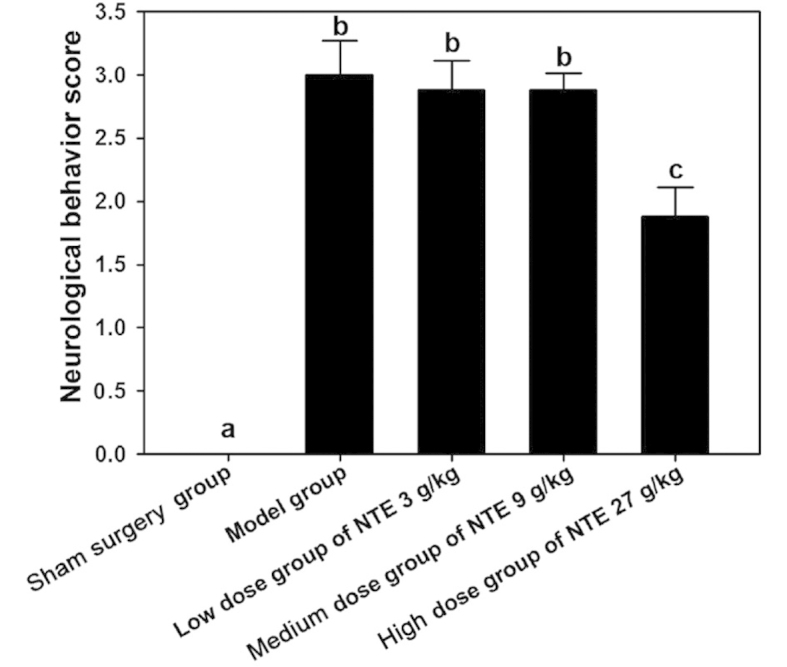
Neurological behavior score in the hippocampal CA2 region following treatment with different doses of NTE. P<0.05 for comparisons between bars labelled with different letters. NTE, naotaifang extract.

Compared with the sham surgery group, the surgery group exhibited a higher neurological behavior score (P<0.05) and compared with the model group, the high-dose group exhibited a lower neurological behavior score (P<0.05; Fig. 3).

**Figure 4:**
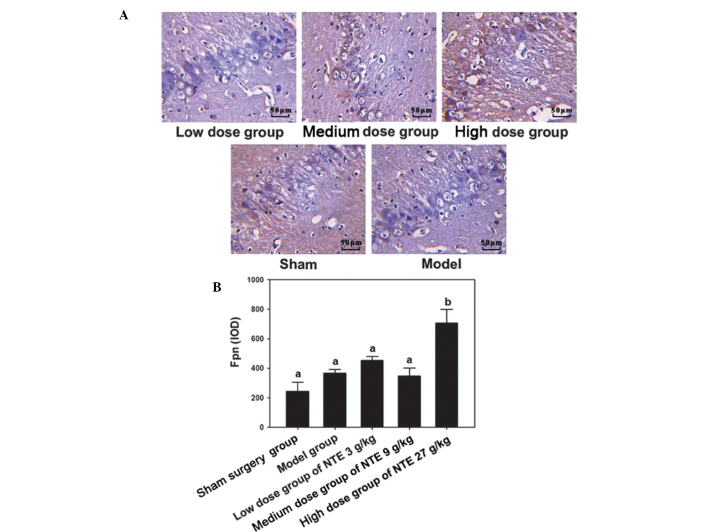
Fpn expression in the hippocampal CA2 area at different doses detected by immunohistochemistry. (A) Immunohistochemistry results of the Sham surgery group, model group, low-dose group (3 g/kg NTE), medium dose group *9 g/kg NTE) and high-dose group 27 g/kg (NTE). (B) IOD of Fpn among all the groups. P<0.05 for comparisons between bars labelled with different letters. Fpn, ferroportin; IOD, integral optical density; NTE, naotaifang extract.

**Figure 5:**
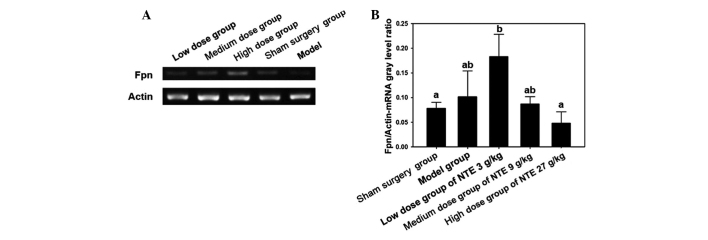
Fpn expression in the hippocampal CA2 area at following treatment with different doses of NTE by RT-PCR. (A) RT-PCR results of the Sham surgery group, model group, low-dose group (3 g/kg NTE), medium dose group (9 g/kg NTE) and high-dose group (27 g/kg NTE). (B) Relative expression of Fpn among all the groups. P<0.05 for comparisons between bars labelled with different letters. Fpn, ferroportin; NTE, naotaifang extract; RT-PCR, reverse transcription polymerase chain reaction.

The immunohistochemical results suggested that following treatment with 3 g/kg NTE, the expression of Fpn increased significantly compared with the other treatment doses (P<0.05). However, no significant changes were observed among other groups (P>0.05; Fig. 4). The RT-PCR analysis also supported the results that only the 12 h treatment group demonstrated a significant increase among all the groups (P<0.05; Fig. 5).

## Discussion

Fpn is a type of non-heme iron exporter protein in the cell membrane. It can be internalized and degraded by binding to hepcidin. The present study demonstrated that expression of Fpn in the hippocampal CA2 region was increased following occlusion of the right MCA, reaching a peak at 12 h, and then decreasing to a minimum at 72 h. The present study demonstrated that intraneural iron metabolism is imbalanced in cerebral ischemia. In addition, the results demonstrate that cellular iron accumulation promotes the expression of Fpn. Iron metabolism imbalance can promote cellular iron efflux. Intracellular iron accumulation may promote the expression of Fpn by a variety of signaling pathways (37). In the present study, following 12 h, intracellular iron efflux increased due to iron overload, however, compensatory adjustment of neurons was limited. After 12 h, the expression of Fpn reduced and iron outflow decreased. The accumulation of iron leads to the increased production of reactive oxygen species and lipid peroxidation, which may damage neurons (38). Thus, it was proposed that if a drug intervention can maintain high Fpn expression following cerebral ischemia, then it may regulate iron metabolism to reduce the damaging effect.

NTE is a traditional Chinese extract that promotes the recovery of neurological function and improvement of blood circulation (39). NTE consists of astragalus root, chuanxiong and dilong. Previous studies have demonstrated that Astragalus may reduce the expression of HIF-1a (hypoxia inducible factor-1a), which protects hippocampal neurons following ischemic brain damage (40). Astragalus may reduce apoptosis in hippocampal neurons through reducing the cellular malondialdehyde and nitric oxide content, thus increasing the activity of superoxide dismutase (41). The active ingredients of chuanxiong contain ligustrazine and ferulic acid (42). Animal experiments have demonstrated that ligustrazine may stimulate neurogenesis following focal cerebral ischemia (43). Previous studies have indicated that ferulic acid may enhance the expression of GABAB1 receptors at 3 h of reperfusion and thereby provide neuroprotection (44). All components in NTE work together in order to promote blood circulation and improve the recovery of neurological function following cerebral ischemia.

Previous studies have demonstrated that Astragalus polysaccharide can prevent neuronal apoptosis following cerebral ischemia (32). Ferulic acid has a neuroprotective effect by promoting the formation of nitric oxide in rat cerebral ischemia (32). Previous studies have verified that NTE is clinically effective for the treatment of cerebral ischemia (45,46). The therapeutic mechanism includes anticoagulation and angiogenesis. Experiment one demonstrated that Fpn expression was reduced to the lowest point 72 h after cerebral ischemia, therefore, in experiment two the animal specimens were produced at the same time point following NTE intervention. The present study demonstrated that a high dose of NTE can increase the expression of Fpn in the hippocampal CA2 region. The traditional Chinese medicine NTE can enhance the expression of Fpn, increase iron excretion in neurons and reduce neuronal oxidative damage caused by iron accumulation. Therefore, the present study concluded that NTE can protect the neurons in the hippocampal CA2 region by increasing the expression of Fpn and promoting neuronal iron efflux in cerebral ischemia.

## Figures and Tables

**Table I:** Nucleotide sequence for oligonucleotide primers of polymerase chain reaction products

Primer	Sequence
Fpn sense	5′-TCCAGTACAGCAGCATCAGCA-3′
Fpn antisense	5′-ACCTCCTTGGGTCCAAACC-3′
Actin sense	5′-CCCATCTATGAGGGTTACGC-3′
Actin antisense	5′-TTTAATGTCACGCACGATTTC-3′

Fpn, ferroportin.
